# Teaching the principle of biological optimization

**DOI:** 10.1186/1754-1611-7-6

**Published:** 2013-02-20

**Authors:** Arthur T Johnson

**Affiliations:** 1 Fischell Department of Bioengineering, University of Maryland, College Park, MD, 20742, USA

**Keywords:** Instruction, Courses, Evolution, Biological systems

## Abstract

Among the important principles in biology that should be taught in biological engineering educational programs is the principle of optimization, what it means, why it is important, and how it comes about. This material can be presented at numerous levels throughout the curriculum. Understanding of this principle can lead biological engineers to expect it in many, if not all, biological system applications. Understanding optimization in biological systems can help understand the predictive power of evolutionary principles and what to expect from living things incorporated in designs.

## Introduction

The paradox of biology is that living things are so energy expensive, even lavish, yet display a certain energy parsimony in almost everything they do. Living things are highly organized internally and externally; they are far from the random assortment of compounds that they would become, and do become once they die, if this organization was not maintained. It takes a great amount of energy to sustain biological organization, whether we consider a single cell, organization of cells within a tissue, tissues within organs, organs comprising an organism, or different individuals interacting in complex ways in an ecosystem.

The only means to overcome the tendency for disorder and increased entropy is a constant supply of energy in form of heat, radiation (sunlight) or chemicals (food or environmental energy-rich compounds). This energy is the means that biological beings and systems survive.

But survival is only the first of two biological imperatives. The second is reproduction amid a (usually) competitive environment. There is a tendency toward domination in the world of biology to make the maximum use of finite resources. Saving operational energy where it is possible allows the organism or system to expend more energy to reproduce. This is, however, usually a zero-sum game. The biological unit that achieves better efficiency than its neighbors has a reproductive advantage and can exploit a larger share of whatever energy resources are available. Although energy is often the most limiting of necessary resources (because of the huge need for energy to sustain biological order), the same can be said for other limited resources, such as food (a source not only of energy but also of essential nutrients), minerals, space, etc.

This tendency towards energy efficiency becomes extreme when the competition extends to the living products of reproduction, the offspring themselves. Hence, the offspring incline to even greater efficiency so that they can compete among themselves.

This applies to biological units (BU) of sundry hierarchical levels and organizations, from individual genes, to cells within a tissue, to organisms, colonies, and ecosytems. Think of the case where there are two BU: one is able to perform the same functions as the second, but it can do so with one half the energy requirements. If each BU is a prey animal, which is most likely to be caught and eaten by a predator? If each BU is a predator, which is most likely to chase and catch an elusive prey, or which is most likely to survive long times between kills? If each BU is a plant, which is most likely to be able to outgrow competitors and grazing herbivores? If each BU is a microbe, which is most likely to inhabit a region with limited nutrient availability? If each BU is a bodily tissue, which is more likely to confer to the entire organism a reproductive advantage? If each BU is a colony of bees, which is more likely to produce more swarms? If each BU is an entire ecosystem, which is most likely to thrive and expand into new territory?

In each of these cases, the answer is clearly that the BU with the advantage in resource utilization is the winner of the competition. Only in the instance where competition is at a minimum, say, for example, for the first species in a virgin environment, will there be little primary reason to reduce energy costs. However, as soon as the second species arrives, or even as soon as the number of individuals of the first species increases to the point that they force significant intraspecific competition, there will be an advantage to those individuals that can make more efficient use of resources. Thus, there is a tendency to minimize dependence upon environmental sources of energy and nutrients.

Natural selection is a powerful force leading to evolution of living things. There is evidence of *convergent evolution* (where organs and tissues with different origins form identical final forms) and *allometric relationships* (scaling of different forms and functions among species). There is enough competitive pressure in biology that the benefit to cost ratio of almost every biological function must be optimized. A hummingbird, for example, needs enough strength in its wings and energy to be supplied to its wing muscles to hover. There is no advantage to be gained with excess wing strength, and so the benefit to cost ratio changes abruptly after sufficient strength is satisfied. On the other hand, animals that jump have a survival advantage if they can jump farther, faster, or higher. There is an overhead cost of supporting larger muscles or bones needed for better jumping, but the benefit to cost ratio changes gradually for jumping. The biologically-optimal solution for these cases would be expected to be different for each.

### In the classroom

An essential part of biological engineering education should be the principles governing typical biological behavior, and, among these principles, biological optimization is one of the most important. Biological engineers should be on the lookout for optima to occur in all aspects of biological activity, and expect optima to define biological states. This is true for everything from locomotion to respiration to neural activity to genetic variability. Each of these will be further discussed later.

Because biological optima are the direct result of competition among living things in an environment with limited resources, and the evolutionary tendency to maximize survival and reproduction, biological engineers who understand how optima appear in biological systems will also better understand the workings of evolution and the ways in which environments are affected by living things and living things affect their environments [1]. Evolutionary principles have been used in powerful ways by biologists, physiologists, and behaviorists to predict biological behaviors. Biological engineers who intend to predict behaviors of living things involved in their designs should thoroughly understand both evolutionary principles, of which optimization is one, and interactions with the environment.

Engineers often translate efficiencies into mathematical optima. If the function to be optimized can be expressed in mathematical form, then simple differential calculus, by setting the first derivative of the function to zero, can be used to find an extremum of that function, as long as the extreme point lies within the boundaries of the problem. There may be local extremes or one global extreme point, and, when discussing these in class, the teacher should be careful to caution students that sometimes the discovery of an optimum depends on the local region around which the extremum is found. This is not too likely to happen with biological systems because then the biological system would exhibit some degree of indeterminacy and that is not very characteristic of living things. It does happen sometimes, but these are often pathological states.

Biological engineering students are characteristically exposed to mathematical models in biology soon after they have taken differential calculus, so some detailed explanations of finding optimum points using the methods they learned in previous calculus courses can be warranted. The teacher should also remind students that extrema may be located at the boundaries rather than in between. This might mean, for instance, that some threshold has been reached, and the instructor can find examples of this to present to the class. For more advanced students, some discussion of means to find optimum solutions for entire functions, such as using calculus of variations, can be included.

At the heart of many biological optimization problems are at least two processes: one that is more costly with increase of the independent variable, and another that is less costly as the independent variable increases. Taken together, there is usually some point where the sum of the outputs from both of these processes is a minimum. This is the optimum point. For biology, investigators have often been interested in energy as the output variable, and have taken as the input variable some entity that makes sense for that particular optimization problem. There are other possible output variables where the maximum becomes the optimum of interest. An example of this might be the efficacy of a certain drug as a function of the time of the day [1,2].

**Figure 1:**
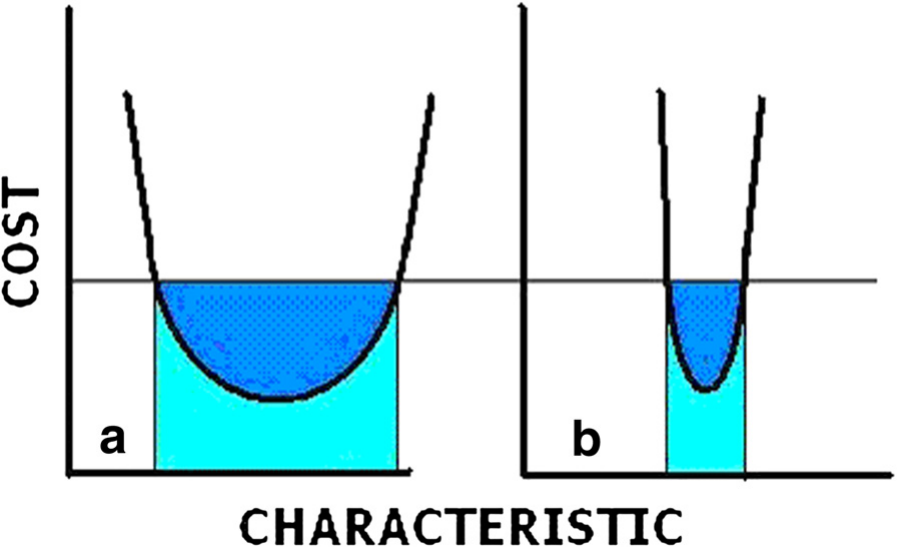
**Illustration of broad (a) optimum and narrow (b) optimum.** For the same level of cost, the broad optimum allows a greater range of the optimized characteristic. Biological systems often seem to prefer broad optima because of the flexibility that they offer.

Optima can be broad or narrow (Figure 1). Narrow optima are very selective, and don’t tolerate much variation before the cost of locating at a nonoptimum point becomes too high to be sustained. An example of this would be the wing strength of the hummingbird just mentioned. In this case, the energy penalty for maintaining wings of excessive strength would not be able to be justified for either survival or reproduction. Broad optima can still have the same optimum, but the costs of deviation from the exact optimum point do not rise significantly quickly. Many biological properties and systems seem to have broad optima. In this case, the advantage is flexibility, allowing deviations that don’t cost too much but that can meet new (environmental) challenges requiring responses different from those determining the original optimum.

Broad biological optima can be a consequence of several properties characteristic of living things. The first is the ability of a living system to sense its environment and to communicate in various ways. The second is the ability to respond to the immediate environment that results in a mathematically chaotic system; that is, the end result is dependent on the pathway it takes to get there. The third property is the appearance of alternate forms that differ very little in energy levels. As a result of these properties, it is easy for biological systems to have varying forms with very little additional energy costs, but which have been determined by the history of the organism.

It is sometimes easier for students to grasp these ideas if biological examples of different optima are presented to illustrate key points. A plethora of examples can also give the impression that optimization is very important in biology and is the result of generations upon generations of evolutionary tendencies. Several examples are given in a succeeding section of this paper to illustrate the range of biological activity seemingly optimized, but the instructor can often add others from personal knowledge or from the current literature.

Biological engineering students who understand the importance of optimization in biology will become more effective in the practice of their profession. They will have known that a biological optimum is located at the preferred operating point, and that deviations from this point can have serious consequences. The engineers are thus likely to make better decisions for their designs involving living things.

### Examples

Optimization can be seen at all biological levels. A few of these are discussed in the following sections.

#### Genetic variability

There is genetic variation within a species that cannot be easily explained. The principle of survival of the fittest (natural selection) should result in the elimination of all but the most survival and reproductively successful genes. This means that genes not optimum for survival in a competitive environment should not persist over many generations, no matter how small their disadvantage. However, a few of them remain, and they give the species the possibility that, should the environment change, there would be genes already present that could be better able to allow the species to adapt correctly.

This can be considered to be an optimization problem; if so, then the unexplained genetic variation found in almost all organisms could be a consequence of the broad optima that characterize biological systems. Therefore, genetic variation could be explained by the fact that carrying nonoptimum genes does not turn out to be too expensive for the species as long as the result of those genes being present does not differ too much from the results of the fittest genes. As with many other biological optima, this means of genetic optimization turns out to be energetically less expensive (and maybe more likely for species survival) than an optimum that confers too much advantage to the best genes.

This can also be illustrated by the presence of the same set of genes in each cell of an organism. Certainly, all the genes present are not activated in every cell, especially if the organization of the organism is highly complex. There should be no need to maintain those genes that are not activated, and the cell expends some excess energy to keep them. There would also be an extra energy expense to tailor a reduced set of genes to each particular type of cell. There is apparently a benefit to cost advantage to the organism to maintain the entire genome in each cell and epigenetically regulate genetic expression.

#### Cellular biochemicals

The activity of many cellular compounds depends on their configurations. This is especially true of proteins and enzymes. Subtle differences in protein folding can have profound effects on effectiveness. Protein misfolding is common, but there are error-correcting schemes within the cell to recycle useless molecules, if possible. Heat shock proteins act as chaperone molecules to reform proteins damaged as a result of heat or other severe environmental stresses. All these mechanisms are designed to recover otherwise worthless molecules and turn them into functioning compounds, thus saving energy and nutrients that otherwise would have been expended by the cell to fabricate these molecules from the start.

As a particularly illustrative example of biomolecule optimization, consider actions of monoclonal and polyclonal antibodies. The former are examples of very narrow functional optima, while the latter are examples of broad functional optima. The biological engineering choice of which antibody type to attach to a biosensor, for instance, depends on how selective the antibody and biosensor is meant to be.

#### Central nervous system

The central nervous system is one of the most energy expensive organs of the human body, requiring about 20% of resting energy expenditure [3]. This energy cost is only indirectly related to survival and reproduction, which are determined directly by physical actions. Optimization applies to the brain, which appears to operate for maximum information transfer among neurons per unit of neural action potential energy used. The two competing functions to form the optimum solution would be: 1) energy maintenance of a number of neurons in an active state, which increases as the number of active neurons increases, and 2) the minimum number of active neurons needed to process required information, a threshold value. Thus, only one to 15 percent of the neurons in the human brain are active at any particular time. Although some would say that using only 15% of the brain’s capacity is not very efficient use of resources, this saves energy for physical movement, such as hunting for food and avoiding predators, that otherwise would have been used for neural activity. Maintaining the presence of extra central nervous system neurons does not cost as much as if they were constantly active, but does result in the flexibility of possible environmental responses.

It has been hypothesized that the timing of human birth optimizes the ability of cognitive and motor neuronal development in the child by allowing the child to maximize the absorption of important cultural information (memes) in its environment [4]. This happens at the expense of the welfare of the child being completely dependent on its caregivers, a condition not shared to the same degree by many other animals. It is also possible that the timing of human birth occurs at the time when the mother’s metabolism is no longer able to support both her needs and the needs of the fetus [4]. In this case, the optimum would be determined by a threshold value instead of the maximum in the case of cognitive development.

#### Walking and running

**Figure 2:**
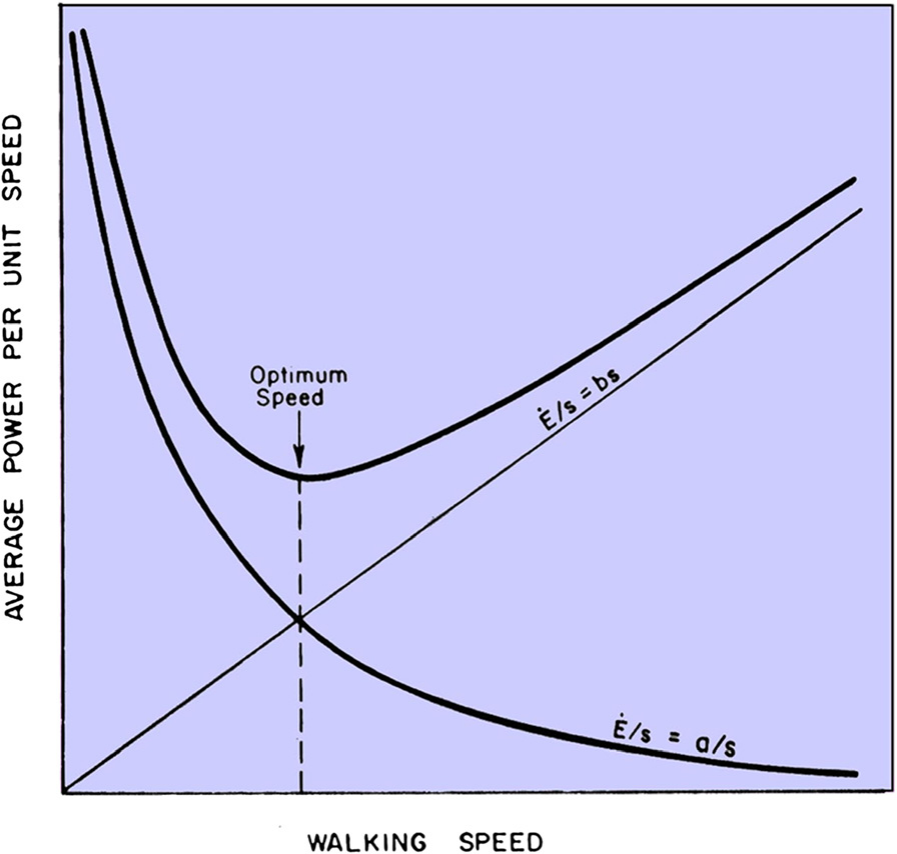
**Walking appears to occur at a speed very close to the optimum based on average power consumption per unit walking speed (E/s).** Two components of average power (E/s), one increasing with speed (bs, where b = a constant) and the other decreasing with speed (a/s, where a = a constant), make possible a minimum average power.

Walking has long been recognized as one bodily function that appears to be optimized, and appears to occur at a speed that minimizes the rate of energy expenditure of the body walking a certain distance (Figure 2). Power consumption of walking, as measured by oxygen consumption, depends on walking speed [5-9]. For any given distance walked, the speed can be higher with extra energy expenditure per unit of time over a shorter total length of time or the energy expended per unit of time can be lower for a longer length of time. The optimum speed of walking is that which minimizes the total amount of energy spent.

Walking is inherently inefficient because at least one foot is on the ground at all times. There are stages when both feet are simultaneously on the ground and pushing against each other [10]. The legs remain nearly straight, and the position of the center of mass of the body is therefore highest when the leg is vertical and the body passes over the supporting foot. Contrarily, the body is lowest when both feet are touching the ground. The body is constantly rising and falling as stepping proceeds [3]. This means that walking comprises a series of episodes of positive physical work when lifting the body mass and negative physical work when the body mass falls, each of these episodes having its own degree of inefficiency. Without optimization, walking would be more inefficient than it is, and so consume too much of the body’s energy resources, making successful survival and reproduction less likely.

Running is a different mode of locomotion in which each foot is on the ground less than half the time. There are stages of running during which neither foot is on the ground. The runner travels in a series of leaps, with the center of mass of the body at its highest in midleap. Its lowest point occurs when the trunk passes over the supporting foot, and the supporting leg is bent at this stage. The running body does not rise and fall as much as during walking, so running can be more efficient at higher speeds.

**Figure 3:**
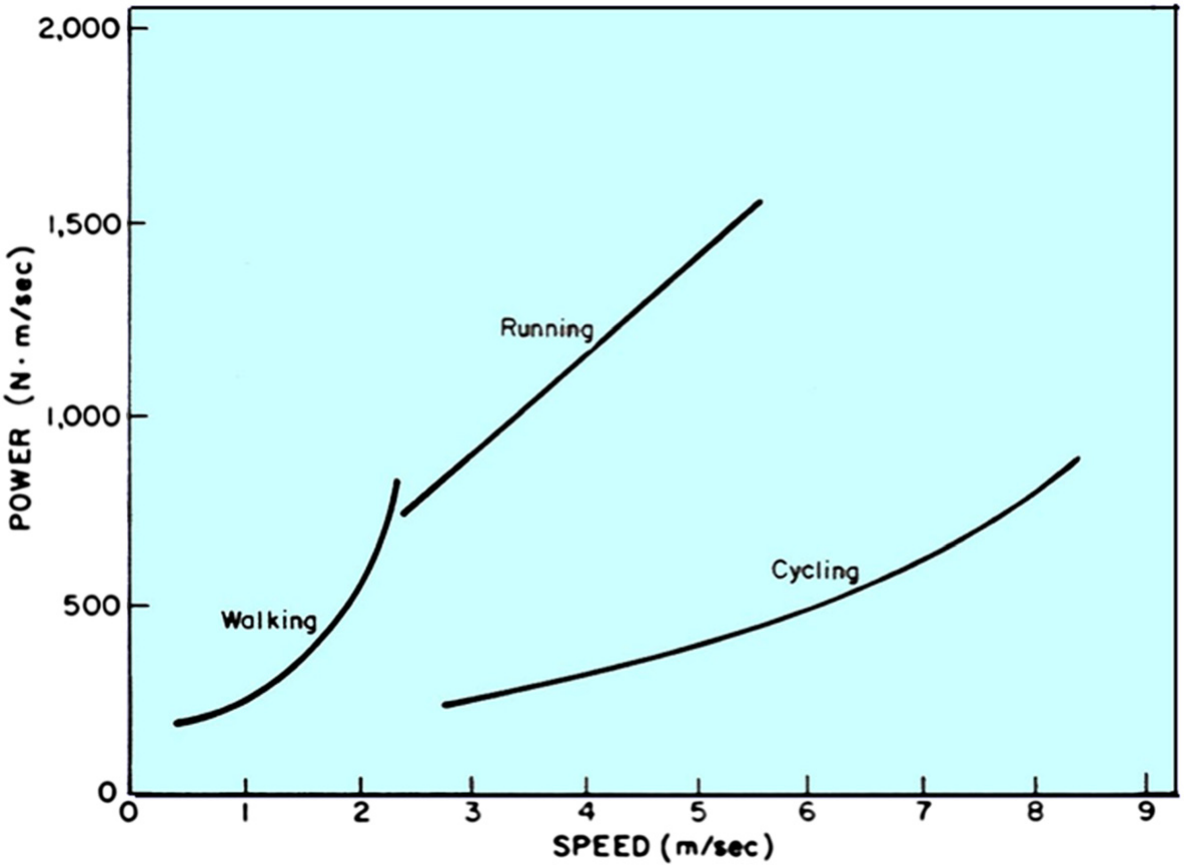
**Power required for walking and running for an adult male human.** Curves for walking and running intersect at about 2.3 m/sec and show that walking is more efficient below the intersection and running is more efficient above.

Caloric measurements have shown that very slow running is more energy expensive than walking at the same speeds, while walking at very high speeds is more costly than running at those same speeds. The transition between walking and running (Figure 3) occurs at a fairly predictable speed of about 2.3 m/sec (6 mi/hr) for normal-sized adults [10], and has been found to occur at the point where the energy expenditures for both walking and running are equal. Again, the energy expenditure of moving about is minimized to make survival and reproduction of the individual more likely than without the minimization.

Quadripedal animals have one mode of locomotion, besides walking and running, that humans do not: they gallop at high speed. Galloping involves bending movements of the back that briefly store leg kinetic energy fluctuations as elastic energy, contributing to overall efficiency [11]. These animals appear to have two transitional power points, one from walking to running or trotting and another from running to galloping.

Other animals use different means to reduce the energy inefficiency of locomotion. Animals such as centipedes have so many legs that their bodies do not rise and fall during locomotion. Snakes crawl without the benefit of legs, but their locomotion must overcome the friction created between the ground surface and their skin. Crocodiles use a side-to-side waddling motion to move their legs forward to propel themselves. Penguins have very short legs that do not raise and lower the body mass by very much. Birds have wings that convert forward motion into lift to improve efficiency. Fishes’ bodies contain a special swim bladder that allows them to maintain a vertical position in the water without muscular effort. It has been estimated that a 1% improvement in the efficiency of a swimming fish can be expected to make 3% more energy available for growth and reproduction [12].

#### Breathing

Human breathing at rest consumes approximately 1-2% of total oxygen consumption of the body, whereas during exercise breathing may consume 8-10% or higher. The oxygen used to breathe is bodily maintenance overhead. Oxygen consumed by the diaphragm and other muscles involved in breathing cannot be used directly by the skeletal muscles in the legs to escape predators, so there should be an evolutionary advantage to save breathing oxygen during heavy exertion.

Breathing at normal frequencies is dominated by resistance located in the airways and lung tissue, and compliance of the lung tissues. As respiration rate increases, so does the rate of airflow and so does the pressure required to push air through the respiratory resistance. Work rate depends on both pressure and flow rate, so the rate of work required to overcome resistance increases nonlinearly with frequency. If the depth of breathing were to be maintained the same at high frequencies as at lower frequencies, then the work rate due to respiratory resistance would be proportional to the square of the flow rate. However, at higher frequencies the alveolar ventilation rate increases above that which is necessary, so flow rate decreases somewhat and work rate is not quite exactly related to flow rate squared.

The pressure required to store energy in a compliance depends upon the volume stored. At higher respiration rates the lungs are not required to fill as much to deliver the same amount of oxygen to the tissues. Thus, compliance pressure, and, consequently work rate, is nearly inverse in magnitude to the frequency increase.

**Figure 4:**
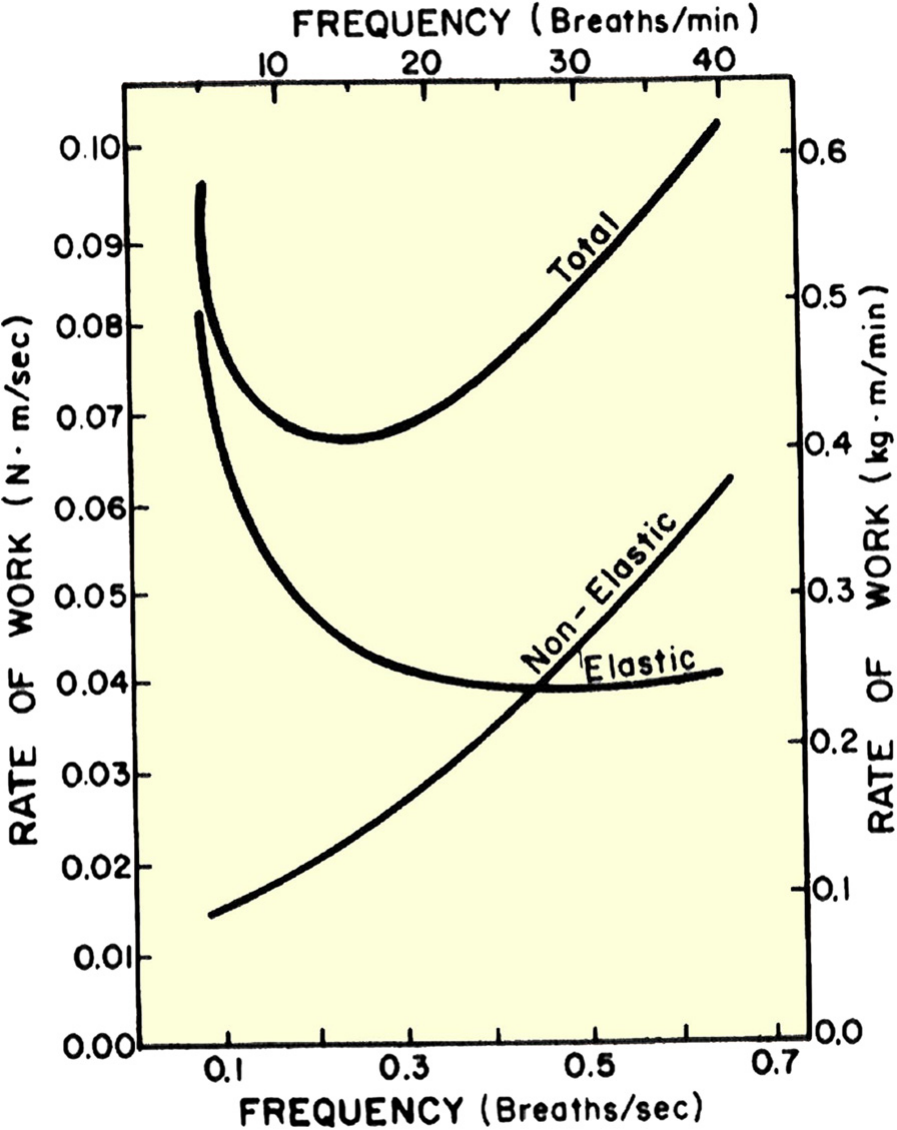
**The work rate of breathing is the sum of resistive (nonelastic) work and elastic (compliance) work.** The sum reaches a minimum at some particular frequency.

The result is that total respiratory work rate is composed of two components, one of which increases with frequency and the other of which decreases with frequency (Figure 4). There is an optimum breathing rate that minimizes work rate, and most published data indicates that people and some other animals breathe at a rate corresponding to the minimum [13]. Exercise breathing is faster because the minimum work rate frequency moves higher as more air is inhaled. Airway caliber, air flow waveshape, ratio of inhalation time to exhalation time, and lung midposition also appear to be adjusted to reduce energy expenditure [3].

#### Heart rate

The work of the heart, similarly to that of the lungs, is necessary to sustain life, but does not perform the work of skeletal muscles necessary to obtain food, escape predators, and survive in other ways. Like respiration, cardiac energy expenditure, although necessary, is part of the overhead used to maintain the health of the physical body. Survival and reproduction of the animal could thus benefit from minimizing cardiovascular work rate.

**Figure 5:**
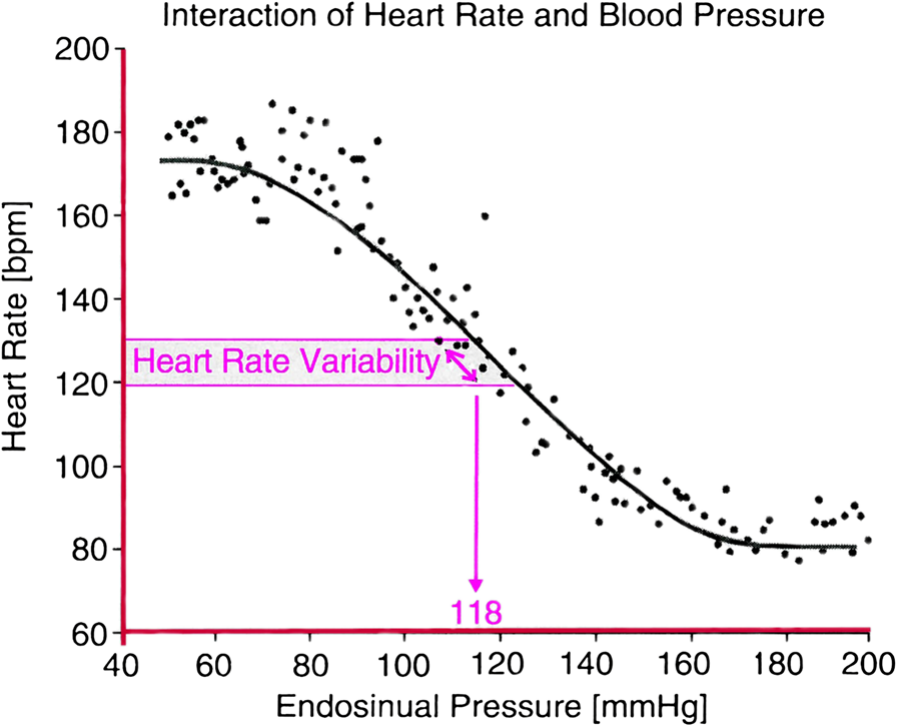
The heart rate is variable, but appears to be located in the most sensitive region to control blood pressure.

Cardiovascular models have shown that it is conceivable that cardiac contraction is performed so as to minimize the rate of work necessary [14]. Variation in heart rate (Figure 5) appears to occur at the most sensitive region to regulate blood pressure [15].

#### Allometry

Allometry is defined as the change of proportions with increase in size of a single species or between adults of related groups [16]. Allometric relations are also known as scaling relationships, and only exist when there is similarity of structure and function between biological units of different size. If completely different mechanisms are involved (for example, locomotion of bacteria compared to horses), then no allometric relationship between them would be expected.

Scaling relationships can be used to predict the value of some biological property of one species if the value of a similar property is known for another species [1]. There appear to be universal biological principles at work in scaling relationships, although the natures of these principles have not yet been fully explored. Allometric relationships among very divergent species seem to be scaled with body mass to some simple multiple of one-quarter power (m^1/4^).

Natural selection seems to have led to an economy of design of structures and functions so that they just meet maximum demands. Any greater capacity would be biologically uneconomical [17]. If evolution results in allometric relationships among BU, then it is only because the benefit to cost ratio of the form or function in question has been optimized. Another way to think of allometry is to consider that, if organisms do *not* change their form as they change in size, their function *is* altered, and such functional shifts might be a source of evolutionary innovation [18].

#### Ecosystems

Entire ecosystems are also models of efficiency. Populations of species that cohabitate these ecosystems complement and supplement each other. Waste from some species is used as nutrition by others; some plants shade others and cool them, and keep them from drying; large trees protect less hardy species from the wind; nutrients that would ordinarily be lost to the ecosystem are stored in the bodies of organisms and recovered when they die.

Many organisms also act as ecosystems home to different varieties of cells, tissues, microbiomes, parasites, and other living things. There is an optimization in this ecosystem to conserve scarce nutrients. Iron, for instance, is recycled from worn-out red blood cells to be used by new red blood cells. The kidney reabsorbs glucose, bicarbonate, some sodium (depending on intake), potassium, and chloride in order to conserve them and reuse them. Desert animals save water by excreting a very concentrated urine.

While these are not exactly the same as optimization that lends itself to mathematical modeling, it is optimization nevertheless. If a more efficient BU is introduced, it likely has a competitive advantage over existing BU, and the less efficient BU is soon displaced. In this way, biological systems are constantly improving their utilization of scarce resources.

### Summary

Understanding the importance of optimization to biological systems is an essential element of biological engineering education [19,20]. Including biological optimization in one or more undergraduate courses can result in better biological engineering graduates who are able to see broad implications for their work. Optimization is a natural result of evolutionary tendencies, so the student who understands the role of optimization in biology can see the workings of evolution as well as the complex interactions of living things with their environments.

## Competing interests

The authors declare that they have no competing interests.

## References

[B1] Johnson AT Biology for engineers 2011 Boca Raton, FL: Taylor and Francis

[B2] Hoven LA Sherman KA Butler S Giebultowicz JM Does the clock make the poison? Circadian variation in response to pesticides PLoS One 2009 4 7 e6469 http://www.plosone.org/article/info../10.1371/journal.pone.000646 10.1371/journal.pone.0006469 19649249 PMC2714471

[B3] Johnson AT Biomechanics and exercise physiology: quantitative modeling 2007 Boca Raton, FL: Taylor and Francis

[B4] Dunsworth HM Warrener AG Deacon T Ellison PT Pontzer H Metabolic hypothesis for human altriciality Proc Nat Acad Sci 2012 109 38 15212 15216 10.1073/pnas.1205282109 22932870 PMC3458333

[B5] Alexander RM Energetics and optimization of human walking and running. The 2000 Raymond pearl memorial lecture Am J of Human Biol 2002 14 641 648 10.1002/ajhb.10067 12203818

[B6] Anderson FC Pandy MG Dynamic optimization of human walking J Biomech Engr 2001 123 5 381 391 10.1115/1.1392310 11601721

[B7] Dean GA An analysis of the energy expenditure in level and grade walking Ergonomics 1965 8 31 47 10.1080/00140136508930772

[B8] Dingwell JB John J Cusumano JP Do humans optimally exploit redundancy to control step variability in walking? PLoS Comput Biol 2010 6 7 e1000856 http://www.ploscompbiol.org/article/info%3Adoi%2F10.1371%2Fjournal.pcbi.1000856 10.1371/journal.pcbi.1000856 20657664 PMC2904769

[B9] Milsum JH Biological control systems analysis 1966 New York: McGraw-Hill

[B10] Alexander RM Walking and running Am Sci 1984 72 348 354

[B11] Alexander RM Why mammals gallop Am Zoolog 1988 28 237 245

[B12] Alexander RM Principles of animal locomotion 2003 Princeton, NJ: Princeton University Press

[B13] Christie RV Dyspnoea in relation to the visco-elastic properties of the lung Proc Roy Soc Med 1953 46 381 386 13055926 10.1177/003591575304600518 PMC1918629

[B14] Livnat A Yamashiro SM Optimal control of left ventricular systolic dynamics Am J Physiol 1981 240 R370 R383 7235053 10.1152/ajpregu.1981.240.5.R370

[B15] Moser M Frühwirth M Kenner T The symphony of life IEEE Eng Med Biol Soc Mag 2008 27 1 29 37 10.1109/MEMB.2007.907365 18270048

[B16] Li JK-J Brown JH, West GB Scaling and invariants in cardiovascular biology Scaling in biology 2000 New York: Oxford University Press 113 128

[B17] Brown JH West GB Enquist BJ Brown JH, West GB Scaling in biology. Patterns and processes, causes and consequences Scaling in biology 2000 New York: Oxford University Press 1 24

[B18] Koehl MAR Brown JH, West GB Consequences of size change during ontogeny and evolution Scaling in biology 2000 New York: Oxford University Press 67 86

[B19] Johnson AT Essential concepts for biological engineers Biol Eng 2010 3 1 3 15

[B20] Johnson AT Making environmental biology central to a course in biology for engineers J Ecosys Ecograph 2011 http://dx.doi.org/10.4172/2157 − 7625.S1-002

